# Fabrication of a visible light photocrosslinkable κ-carrageenan-gelatin hydrogel patch to aid in wound healing applications

**DOI:** 10.3389/fbioe.2026.1846548

**Published:** 2026-08-05

**Authors:** Menaga S, Sushma Kumari

**Affiliations:** 1 Centre for Biomaterials, Cellular and Molecular Theranostics, Vellore Institute of Technology, Vellore, India; 2 School of Bio Sciences and Technology (SBST), Vellore Institute of Technology, Vellore, India

**Keywords:** chemical modification, gelatin, kappa carrageenan, tissue engineering, visible light photocrosslinking, wound healing

## Abstract

A delay in wound healing significantly impacts a patient’s quality of life. Although numerous materials have been developed for healing strategies, there remains a need to create a patient-friendly, patient-specific, and biocompatible product for the market. Biopolymer-based hydrogel dressings significantly influence wound healing by mimicking the extracellular matrix, promoting cell growth, regulating moisture levels, and preventing infection. In this study, biopolymers such as κ-carrageenan (κ-Ca) and gelatin (Gel) were chemically modified with methacrylic anhydride (MA) to produce photocurable κ-carrageenan methacrylate (κ-Ca-MA) and gelatin methacrylate (Gel-MA). A hydrogel patch was subsequently fabricated using visible-light photocrosslinking for wound-healing applications. The fabricated photocrosslinked composite hydrogel patch (PCHP) was comprehensively characterized with respect to its morphological structure, swelling, degradation behavior, and viscoelastic properties. The fabricated PCHP demonstrated gel-like behavior, good stability, and shear-thinning properties. Compression testing on PCHP revealed a compressive modulus of 234–239 kPa, demonstrating adequate mechanical integrity for wound-site application. Additionally, antibiotic-loaded PCHPs demonstrated slow, sustained antibiotic release with efficient bactericidal activity against *Escherichia coli* and *Staphylococcus aureus*, as well as notable radical-scavenging activity. Scratch assays demonstrated effective promotion of wound healing through enhanced cell migration, resulting in complete wound closure within 24 h. NIH3T3 fibroblasts cultured on PCHPs showed greater than 95% viability and cell proliferation over 5 days. Furthermore, κ-Ca-MA- and Gel-MA-based hydrogels were suitable for 3D printing in a layer-by-layer manner using digital light processing (DLP), highlighting their potential for advanced biomedical applications. Therefore, the visible-light photocrosslinking approach enables the creation of a multifunctional product for wound-healing applications, thereby improving patients’ quality of life.

## 1 Introduction

Skin, as the foremost barrier of our body, protects us from microbial infections and the harsh external environment (Lin et al., 2025). A wound refers to any disruption of the skin brought about by physical, chemical, or heat-induced damage. Healing time and wound depth categorize wounds into acute or chronic. Acute wounds heal within 4 weeks through the person’s immune system. Conversely, chronic wounds persist for more than 6 weeks with delayed healing due to factors such as the patient’s age, reduced mobility, and medical complications like vascular disease and diabetes. When skin is damaged, our body initiates a cascade of reactions to heal the wound. Healing of a chronic wound involves multiple interconnected four stages: hemostasis, inflammation, cell proliferation, and remodeling of the extracellular matrix (ECM) to reconstruct the tissues and cells at the damaged site. The primary concern in chronic wound healing is to prevent microbial invasion and biofilm formation, maintain moisture, and remove exudates from the wound area (Gounden and Singh, 2024; Deng et al., 2022; Uberoi et al., 2024).

Several wound-healing materials, including injectable hydrogels, films, 3D-printed constructs, electrospun fibrous mats, and various polymeric materials, have emerged for wound-dressing applications (Pourmadadi et al., 2025). However, establishing an optimal microenvironment for wound healing and preventing microbial infections in chronic wounds remains a significant challenge. Therefore, there is a need to develop user-friendly biomaterial-based 2D and 3D novel materials and fabrication processes to overcome existing problems, such as removing exudates, maintaining wound area moisture, maintaining a neutral pH, protecting against microbial invasion, and promoting faster skin regeneration (Chakraborty et al., 2023; Freedman et al., 2023).

Polymers have made notable contributions in tissue engineering and regeneration over recent decades, owing to their higher biocompatibility and widespread availability. In the biomedical field, natural biopolymers have been more widely explored for biomedical engineering applications than synthetic polymers, due to factors such as renewability, compatibility, degradability, and ECM-mimicking properties (Tavakoli et al., 2023; Sheokand et al., 2023; Elango et al., 2023; Gruppuso et al., 2021; Gobi et al., 2021; Jaiswal and Sherje, 2024; Zhang et al., 2024). Over the last several years, researchers have used polysaccharide-based films, sponges, hydrogels, and 3D bioprinted scaffolds in biomedical engineering owing to their greater biocompatibility and potential to mimic the ECM (Tchobanian et al., 2019). In prior research, polysaccharide-based three-dimensional networks have been utilized in diverse biological and healthcare applications by employing various crosslinking strategies, including physical, chemical, enzymatic, and photocrosslinking methods, to enhance their mechanical and morphological properties (Liu et al., 2025). In physical crosslinking, UV and microwave-assisted irradiation can cause cytotoxicity and cell damage, thereby limiting cell proliferation and attachment. Meanwhile, in the chemical crosslinking strategy, crosslinking agents such as glutaraldehyde, genipin, and carbodiimide may not be fully removed by washing and can remain in the scaffold, potentially inducing cytotoxicity and other chemical reactions when used for biomedical applications. In enzymatic crosslinking, crosslinkers such as transglutaminase (TGase) and microbial transglutaminase (mTGase) are expensive, exhibit substrate specificity, and may induce immunogenic reactions. Recently, photocrosslinking has emerged as a promising strategy to fabricate 3D biomaterials with controlled structure. Here, by attaching light-responsive functional groups, the biomaterial becomes photocurable, enabling the fabrication of 3D scaffolds with minimal photoinitiator under light exposure (Choi et al., 2019; Moon et al., 2023; Li et al., 2023). In contemporary studies, many researchers have successfully demonstrated the use of blue light (400–495 nm) in fabricating 3D scaffolds and have shown its non-cytotoxicity, biocompatibility, and suitability for medical applications (Kowsari et al., 2021; Menon et al., 2024; Waqar et al., 2025).

In this research paper, a visible-light photocrosslinking strategy is employed to produce the desired product. As the extracellular matrix (ECM) is rich in glycosaminoglycans (GAGs) and proteoglycans, we selected biopolymers such as κ-carrageenan (κ-Ca) and gelatin (Gel) to replicate the ECM’s structure and function (Hachim et al., 2019; Jabeen et al., 2025; Sonwane et al., 2025; Echave et al., 2017). Both biopolymers exhibit shear-thinning behavior, thermoresponsiveness, and non-cytotoxicity and biocompatibility. Here, we chemically modified the polysaccharide (κ-Ca) and protein (Gel) with methacrylic functional groups to make them photocurable biomaterials. κ-Ca-MA and Gel-MA were mixed with a minimal amount of photoinitiator and exposed to visible light at 420 nm to form the photocrosslinked composite hydrogel patch (PCHP). This noncytotoxic photo-crosslinking strategy, which involves covalent bonding, enhances the biomaterial’s cytocompatibility and facilitates achieving mechanical stability to simulate *in vitro* stiffness and biocompatibility. The fabricated photocrosslinked composite hydrogel patches (PCHPs) were intensively characterized for swelling, degradation, viscoelastic, and mechanical properties. To achieve targeted antibacterial effects, PCHP were loaded with antibiotics and investigated for antimicrobial studies. To assess biocompatibility, NIH3T3 fibroblasts were seeded into PCHP and analyzed for cell adhesion and proliferation. We also investigated the wound healing potential of the PCHPs using a scratch assay. Furthermore, hydrogels have been demonstrated for use in 3D printing, specifically digital light processing (DLP), to fabricate patient-specific 3D scaffolds.

## 2 Materials and methods

### 2.1 Materials

Methacrylic anhydride, gelatine from porcine skin with gel strength of 300 (Type A), dialysis tubing (MWCO: 12–14 kDa), lithium phenyl (2,4,6-trimethyl benzoyl) phosphinate (LAP), Diphenyl-1-picrylhydrazyl (DPPH), and resazurin were purchased from Sigma-Aldrich. Kappa carrageenan (κ-Ca) was sourced from Tokyo Chemical Industry (TCI). Dulbecco’s Modified Eagle Medium (DMEM), phosphate-buffered saline (PBS), antibiotic-antimycotic (100X), fetal bovine serum (FBS), trypsin, gentamycin, kanamycin, tetracycline, nutrient agar, Luria broth (LB), Crystal violet (CV), Muller-Hinton broth, and DMSO were obtained from Hi-Media. In all experiments, we used deionized water from the Milli-Q water purification unit (Millipore).

### 2.2 Chemical modification of κ-carrageenan with methacrylate groups (κ-Ca-MA)

κ-Ca was chemically modified to κ-Ca-MA using the previously reported protocol (Kumari et al., 2022). Concisely, 1% w/v (500 mg) of κ-CA powder was dissolved in 50 mL of Milli-Q water and stirred at 55 °C. After complete dissolution of the κ-Ca powder, 6 mL of methacrylic anhydride was added to the reaction mixture using a dropper. The pH was adjusted to 8 with 4.0 M NaOH, and stirring was performed for 6 h at 55 °C. After the completion of the methacrylate substitution process, the solution was brought to room temperature and transferred to a 12–14 kDa dialysis tube. The solution was dialyzed against Milli-Q water continuously for 4 days to remove the unreacted methacrylate and other by-products. The solution was stored in a sterile container at −80 °C for pre-freezing, then lyophilized for 6 days. The lyophilized κ-Ca-MA was preserved at −20 °C for subsequent experiments.

### 2.3 Chemical modification of gelatin with methacrylate groups (Gel-MA)

The methacrylate modification of gelatin was carried out with slight modifications (Sun et al., 2018; Chen et al., 2023). Here, 10% w/v gelatin was dissolved in 30 mL PBS at 50 °C under continuous stirring at 500 rpm. After dissolving the gelatin, 6 mL of methacrylic anhydride was added dropwise with continuous stirring for 1 h. PBS was added four times to the methacrylate gelatin solution to stop the reaction. Then, the synthesized Gel-MA solution was transferred to a 12–14 kDa dialysis tube and dialyzed against Milli-Q water for a week. The dialyzed Gel-MA solution was pre-frozen at −80 °C, followed by lyophilization for 3–4 days. All the lyophilized Gel-MA was stored at −20 °C for subsequent experiments.

### 2.4 Ink optimization to develop a composite patch for visible light photocrosslinking

Lyophilized samples of both κ-Ca-MA and Gel-MA were dissolved in PBS with varied concentrations from 1% (w/v) to 5% (w/v) and kept at 40 °C for complete dissolution. The completely dissolved solution was mixed with 0.4% LAP photoinitiator and exposed to visible light at 420 nm in a Herber’s multi-UV curing photoreactor for 20 s to fabricate the PCHP.

### 2.5 Field emission scanning electron microscopy (FE-SEM)

The morphology and porosity of the PCHP were visualized using FE-SEM at an accelerated voltage of 10 kV. To analyze the sample, PCHP of κ-Ca-MA and Gel-MA were lyophilized and mounted on an Aluminum stub. The stub was then sputter-coated with gold, and the image was captured at various magnifications (150x-1300x) using a QUANTA FEG-250 at 10 kV.

### 2.6 Fourier transform infrared spectroscopy (FTIR)

Lyophilized samples of κ-Ca, κ -Ca-MA, Gel, and Gel-MA were used for FTIR interpretation in the ATR (Attenuated total reflectance) mode. To confirm the functional groups, we performed FTIR spectroscopy on a Thermo-Nicolet iS50 spectrometer, acquiring spectra from 4000 to 500 cm^-1^.

### 2.7 NMR (nuclear magnetic resonance) spectroscopy

We measured the ^1^H NMR spectra of the samples (κ-Ca, κ-Ca-MA, Gel, and Gel-MA) dissolved in D_2_O to analyze the chemical structure and quantify the degree of methacrylation using a Bruker NMR spectrometer at 400 MHz.

### 2.8 Rheology measurements

PCHP were tested using a 25 mm (diameter) flat parallel plate with a 1 mm gap at room temperature, 26 °C. The shear rates of PCHPs were recorded from 0.01 to 100 s^-1^ for viscosity tests. An amplitude sweep covering shear strains from 0.1% to 1000% was performed at 10 rad/s to characterize the viscoelastic behavior of the material. Furthermore, at a fixed strain of 0.5%, a frequency-sweep test was conducted over the range 0.1–100 rad/s using an Anton Paar Compact Rheometer-92 to determine the viscoelastic characteristics.

### 2.9 Mechanical testing

The compressive stress-strain curves for all samples having 1.5 cm × 1.5 cm dimensions with different PCHP concentrations were analyzed using Universal Testing Machine (H5KS, Tinius Olsen Ltd., United Kingdom) equipped with a 50 N load cell. The crosshead displacement rate was fixed at 2 mm/min, and the specimens were compressed to 70% strain. Data acquisition and analysis were performed using Horizon software.

### 2.10 Swelling test

PCHP was further analyzed for its swelling properties. We measured the pre-swelling dry weight (W_dry_) of the PCHP and subsequently immersed it in PBS solution for 24 h at room temperature. PCHP were removed at different time intervals to measure swelling weight (W_t_). The swelling ratio of the visible light-crosslinked hydrogel patch was calculated using the following equation:

$$Swelling\;ratio=\frac{W_{t}-W_{dry}}{W_{dry}}$$

### 2.11 Porosity

The ethanol filtration method was used to determine the scaffolds’ porosity (Pan et al., 2017). Briefly, PCHPs with dry weight (W_dry_) were immersed in absolute ethanol for 5 h at room temperature. After the stipulated time, PCHPs were removed from the ethanol solution, the excess ethanol was blotted with filter paper, and the infused scaffold weight (W_1_) was recorded. Finally, the porosity was calculated using the following equation:

$$\text{Porosity }\left(\%\right)=\frac{W_{1}-W_{dry}}{\rho_{ethanol}\times V_{s}}\times 100$$

Here, W_1_ is the ethanol-saturated scaffold weight, W_dry_ is the lyophilized scaffold weight, ***ρ*** is the density of ethanol, and V_s_ will be the geometric volume of the scaffold.

### 2.12 Degradation test

At first, the dry weight of lyophilized PCHP was measured and taken as (W_dry_), followed by soaking in DMEM at room temperature. At various time intervals from day 1 to day 15, the patch was removed, followed by three deionized water rinses and lyophilization to determine its non-degraded weight (Wt). We calculated the degraded weight using the following equation:

$$Degraded\;weight\;\left(\;\%\right)=\frac{W_{dry}-W_{t}}{W_{dry}}\times 100$$

### 2.13 Direct antibiotic loading and release study

Gentamycin (600 µg), kanamycin (600 µg), and tetracycline (300 µg) were incorporated using the physical entrapment method. The antibiotics were mixed with a solution of κ-Ca-MA and Gel-MA, then crosslinked under visible light at 420 nm in the presence of 0.4% LAP photoinitiator.

The absorbance of standard gentamicin, kanamycin, and tetracycline solutions was first measured at 202 nm, 206 nm, and 364 nm, respectively, to prepare the calibration curves for drug release analysis. The calibration curves showed good linearity between concentration and absorbance, with *R* ^2^ values of 0.995, 0.997, and 0.998 for gentamicin, kanamycin, and tetracycline, respectively. To evaluate the release profile, antibiotic-loaded PCHP samples were immersed in phosphate-buffered saline (PBS) at 37 °C. At predetermined time intervals, the entire volume of PBS containing released antibiotics was removed and replaced with an equal volume of fresh PBS. The concentration of antibiotics in the collected solution was quantified by UV spectrophotometry and calculated using the following equation below:

$$Drug\;release\left(\%\right)=\frac{Cumulative\;drug\;released\;with\;time}{Total\;drug\;loaded}\times 100$$

### 2.14 *In vitro* antibacterial assay

The PCHP were tested against bacteria to determine their efficacy in inhibiting growth and to measure colony-forming units and zone of inhibition. Because the chronic wound is polymicrobial, this work specifically selected one Gram-positive and one Gram-negative bacterium for the experiment. *Staphylococcus aureus* (ATCC 25923) and *Escherichia coli* (ATCC 25992) strains were used for antibacterial assays, as they are the predominant species in the chronic wound environment.

- Colony-forming unit: The colony-forming unit (CFU) was assessed by incubating the drug-loaded and non-loaded PCHP films with 10 mL of a 1:50 bacterial suspension (OD_600_ of 0.1) and broth for 12 h at 37 °C. The resulting incubated bacterial culture was diluted to 100 µL and inoculated onto a nutrient agar plate, which was then left undisturbed for 24 h (Li et al., 2022). After a day, images of the bacterial colonies were captured with a smartphone, and the scaffold without antibiotics served as the control. The CFU was calculated based on the following equation:

$$CFU=\frac{Number\;of\;colonies\times Dilution\;factor}{Volume\;plated}$$

ii. Zone of inhibition (Kirby Bauer test): We cultured bacterial cells such as *S. aureus* and *E. coli* (OD_600_ of 0.1) onto the agar plates through the spread plate technique. The PCHP loaded with antibiotics (gentamycin, kanamycin, and tetracycline) were carefully placed on agar plates and incubated for 24 h at 37 °C in the incubator to assess their potential to inhibit bacterial cell growth. The non-loaded patch served as the control in this study. After 24 h, the plates were removed, and the zone of inhibition was measured as the diameter of the clear zone surrounding the scaffold (Hudzicki, 2009).
iii. Bacterial biofilm inhibition assay: A crystal violet assay was performed to assess biofilm inhibtion (Fasiku et al., 2021). Briefly, the prepared hydrogels, such as PCHP-G, PCHP-K, PCHP-T, and PCHP, were UV-sterilized and incubated with 1 mL of a 0.1 OD bacterial suspension for 24 h. After 24 h of incubation, the gels were washed 3–4 times with PBS at pH 7.4 to remove non-adherent cells. Further, the washed gels were stained with 0.1% of crystal violet and incubated for 15–20 min. The crystal violet dye was removed after 15–20 min, and the hydrogels were washed multiple times with PBS to remove excess stain. Finally, the gels were treated with 33% glacial acetic acid to solubilize the bound crystal violet and incubated in a shaking incubator for 10–15 min. The optical density was recorded with spectrophotometry at 570 nm. The antibiofilm activity was calculated using the following equation:

$$Antibiofilm\;activity\;\left(\%\right)=\frac{{OD}_{control}-{OD}_{antibiotic\;treated}}{{OD}_{control}}\times 100$$

iv. Bacterial viability: The Alamar blue assay was performed to detect bacterial cell metabolic activity *via* the dye’s reduction reaction. The bacterial cells of both *E. coli* and *S. aureus* were inoculated into nutrient broth and incubated overnight at 37 °C. The incubated bacterial suspensions were adjusted to 10^6^ CFU/mL (OD_600_ 0.08–0.1). Then, 100 µL of bacterial suspensions and 100 µL of the patch extracts were plated in a 96-well plate and incubated for 24 h under static conditions. Here, wells containing bacterial cells and nutrient broth alone serve as the positive control. After 24 h, the samples and control were treated with 10% (v/v) Alamar blue reagent and incubated for 2 h. Fluorescence readings were taken with a Tecan microplate reader at 570 nm.
v. Bacterial attachment for SEM analysis: A bacterial attachment assay was performed with *E. coli* and *S. aureus.* Briefly, the scaffolds of control and loaded samples were immersed in 1 mL of a bacterial cell suspension (OD600 of 0.08–0.1) for 24 h at 37 °C. Then, the bacterial suspensions were decanted and washed 5–6 times with PBS, followed by a sterile water wash identical to the PBS wash. Followed by glutaraldehyde fixation with 2.5% for 30 min at 37 °C. The scaffolds were sequentially dehydrated in graded ethanol solutions (10%, 30%, 50%, 70%, and 100%) and imaged by SEM after gold sputter coating.

### 2.15 *In vitro* antioxidant activity

The ROS scavenging activity of the antibiotic-loaded PCHPs was assessed using the diphenylpicrylhydrazyl (DPPH) dye in a direct-contact method (Thi et al., 2020). PCHPs were incubated with 2 mL of 0.1 mM DPPH solution for 30 min in dark, followed by UV absorbance measurement at 517 nm. The DPPH solution was taken as a control and scavenging (%) was calculated using the following equation:

$$\text{DPPH scavenging }\left(\%\right)=\frac{Control-Treated}{Control}\times 100$$

### 2.16 *In vitro* assays

- Live/Dead cell assay: NIH 3T3 fibroblasts (National Centre for Cell Science, Pune, India) were seeded onto PCHP scaffolds and incubated for 24 h in DMEM supplemented with 10% fetal bovine serum and 1% (v/v) antibiotic-antimycotic solution under standard culture conditions (37 °C, 5% CO_2_, 95% humidity). Prior to seeding, cell viability and density were determined using trypan blue exclusion in a Neubauer chamber. 2 μM Calcein acetoxymethyl ester (Calcein AM, Invitrogen) and 4 µM Ethidium Homodimer-1 (EthD-1, Invitrogen) were used for staining fibroblasts as live and dead, respectively. After 45 min, PCHP was gently rinsed twice with PBS to remove the excess stain. Fibroblasts on PCHP were scanned and imaged through a confocal microscope (Olympus Fluoview FV3000 with CellSens software) with excitation channels set to red and green. The data were processed using ImageJ.
- Proliferation assay: We performed an Alamar Blue assay (resazurin reduction) to evaluate the cytotoxicity of PCHPs seeded with NIH 3T3 fibroblasts over 5 days. At each observation day, media were removed from the test samples. A 10% (v/v) resazurin solution (100 μg/mL) prepared in fresh cell culture medium was added to the test samples, followed by a 3-h incubation at 37 °C. Using a microplate reader (Tecan), the metabolic reduction of resazurin, a blue, non-fluorescent dye, to resorufin, a pink fluorescent product, was monitored with an excitation filter at 530 nm and an emission filter at 600 nm.
- Scratch assay: NIH3T3 cell lines were chosen and seeded in a 35 mm cell culture plate, allowing for confluency. After the cells reached confluence, the media in the cell culture dish was decanted, and a small scratch was made to mimic the wound model using a 200 µL sterile tip. Furthermore, they were given a PBS wash to remove the detached cells during the scratch procedure. The extract of the photocured hydrogel, loaded with drugs and without drugs, was subsequently introduced to the scratch-induced monolayer cell culture plate. The plate was analyzed under a Olympus microscope at 0, 6, 12, and 24 h to assess cell migration using the CellSens application (Liang et al., 2007). Migration of NIH3T3 cells was quantified from scratch assay images using ImageJ software. The initial wound area (A_0_) was measured at time zero, and the wound area at subsequent time points (A_t_) was measured as the gap width at time t. The percentage of wound closure was calculated using the following equation:

$$Wound\;closure\;\left(\;\%\right)=\frac{A_{0}-A_{t}}{A_{0}}\times 100$$

### 2.17 DLP printing

DLP printing was performed using LUMEN X GEN 3, with a Cellink setup featuring 35 µm pixel resolution, a build volume of 68 × 38 × 100 mm, and a temperature range from room temperature to 60 °C. The setup also included an LED wavelength of 402 nm, an intensity range of up to 30 mW/cm^2^, UV-C sterilization, and a 70 × 45 mm (large) glass-bottom build platform. Structures, such as circles and squares, with different dimensions (8 × 8 × 2) and 20 layers, were used for 3D printing. PCHP fabrication involves printing a precursor composition of photocurable κ-Ca-MA and Gel-MA, 0.4% LAP, and 0.3 mM tartrazine, and exposing each layer to 405 nm light to polymerize the solution. The DLP printing parameters were set to 50 μm, a curing time of 20 s, and a 70% projector power level at room temperature. The printed structures were further rinsed with PBS and stored under appropriate conditions until further use.

### 2.18 Statistical analysis

All the experiments were performed in triplicate (*n* = 3) and results are expressed as means ± standard deviations (SD). All the statistical analysis were done using Origin pro (2026 Version). One way analysis of variance (ANOVA), followed by Tukey’s *post hoc* test was used to determine the significance difference among groups. A *p*-value of <0.05 was considered statistically significant.

## 3 Results and discussion

### 3.1 Methacrylation of κ-Ca-MA and Gel-MA

**FIGURE 1:**
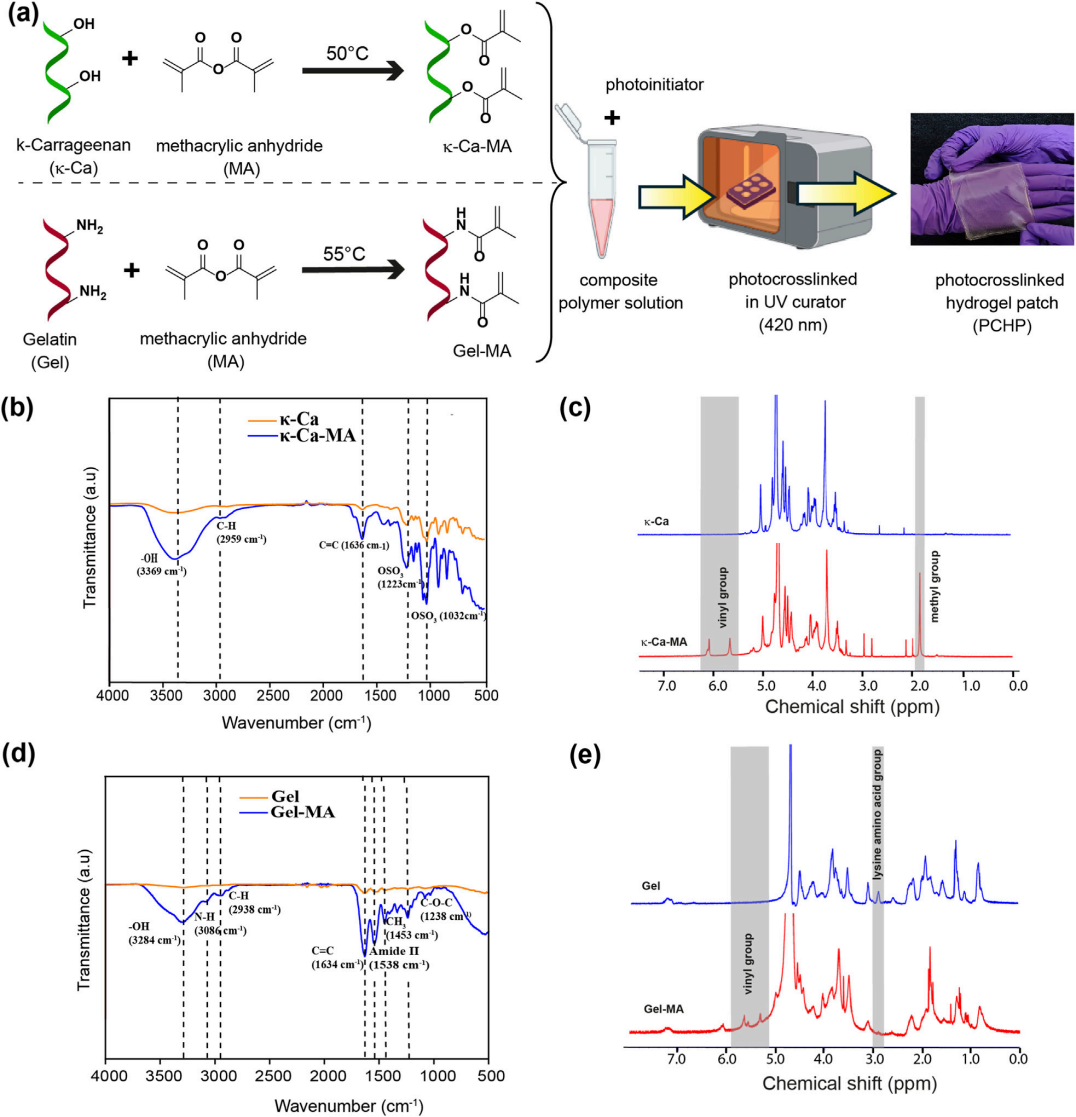
**(a)** Schematic illustration showing the synthesis of photocurable biopolymers, where κ-carrageenan (κ-Ca) and gelatin (Gel) were chemically modified to form methacrylated derivatives κ-Ca-MA and Gel-MA, **(b)** FT-IR analysis of κ-Ca and κ-Ca-MA, **(c)** NMR analysis of κ-Ca and κ-Ca-MA, **(d)** FT-IR analysis of Gel and Gel-MA, and **(e)** NMR analysis of Gel and Gel-MA.

To prepare photocurable polymers, κ-Ca and Gel were functionalized with a methacrylic anhydride solution, as described earlier (Kumari et al., 2022; Kumari et al., 2024). Methacrylic anhydride solution is an organic monomer used to chemically functionalize polymers’ hydroxyl groups, enabling photocrosslinking upon exposure to light at 420 nm with blue light (Figure 1a). Methacrylate-functionalized polymers were analyzed both qualitatively and quantitatively to identify their functional groups. In the FTIR data of κ-Ca-MA, a double-bond peak (C=C) at 1636 cm^−1^ was observed, which was distinct from the FT-IR spectrum of unmodified κ-Ca (Figure 1b). For quantitative analysis, ^1^H NMR of κ-Ca-MA supported the presence of the methacrylate functional group through the identified peaks of the vinyl protons (-CH_2_) and methyl group (-CH_3_) at 6.2 and 5.7 ppm (doublet peak), 2.0 ppm (singlet peak), respectively (Figure 1c). These peaks assigned to the methacrylate functional group were not observed in the ^1^H NMR spectrum of κ-Ca NMR, confirming the successful functionalization of κ-carrageenan with methacrylic anhydride. A degree of substitution (DS) of 33.3% was evaluated through comparison of the average integral values of the vinyl proton signals (δ = 5.5–6.4 ppm) to that of the anomeric proton (δ = 5.1 ppm) from the 3,6-anhydro-α-D-galactopyranose residue present in κ-carrageenan (Mihaila et al., 2013; Tavakoli et al., 2019).

FT-IR and ^1^H NMR spectroscopy confirmed the methacrylation of Gel (Yan et al., 2022; Golshirazi et al., 2025). The FTIR spectra of Gel-MA displayed characteristic absorption peaks, including the double bond C=C at 1634 cm^-1^, CH3 at 1453 cm^-1^, C-O-C at 1238 cm^-1^, hydroxyl group at 3,284 cm^-1^, N-H at 3,086 cm^-1^, and C-H at 2,938 cm^-1^, as shown in Figure 1d. Additionally, Figure 1e shows that the ^1^H NMR spectrum of Gel-MA exhibited a characteristic vinyl proton signal at approximately 5.3 and 5.6 ppm, along with a reduced lysine peak at 3.1 ppm, which was absent in non-methacrylated Gel. The ^1^H NMR data confirm successful methacrylation, and the degree of methacrylation was quantified as 30% using a previously reported formula (Golshirazi et al., 2025).

### 3.2 Preparation of composite patch

Photocurable synthesized κ-Ca-MA and Gel-MA polymers were dissolved in PBS at varying amounts (1%–3% w/v for κ-Ca-MA and 2.5%–4.5% w/v for Gel-MA) and were optimized for photocrosslinking with 0.4% LAP (Figure 1a). The polymeric solution was irradiated with visible light at 420 nm for 20 s to initiate photopolymerization, yielding a 3D photo-crosslinked hydrogel patch (PCHP) with strong covalent bonds, as confirmed across a range of concentrations. However, PCHPs composed of 2.5% (w/v) κ-Ca-MA and 2.5%–4.5% (w/v) Gel-MA were selected for all subsequent studies and analyses.

### 3.3 Morphology of the photocured patch

**FIGURE 2:**
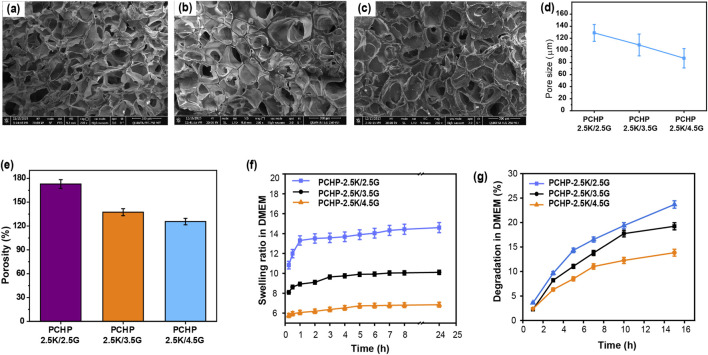
**(a–c)** SEM images of the PCHP-2.5K/2.5G, PCHP-2.5K/3.5G, PCHP-2.5K/4.5G, and **(d)** corresponding pore size distribution calculated using ImageJ software. Scale bar: 300 µm. **(e)** Porosity percentage of different PCHPs based on hydrogel scaffolds evaluated using the ethanol displacement method. **(f)** The water uptake profile, and **(g)** degradation profile of the PCHPs measured in cell culture media DMEM at different time intervals. Data are presented as mean ± SD (n = 3).

We performed FE-SEM analysis of lyophilized PCHPs to evaluate the morphology, topography, and interconnectivity. As shown in Figures 2a–c, the structural analysis from SEM images revealed that all the PCHPs have high, interconnected porosity and thick walls, making them suitable for biomedical applications such as wound-healing or tissue-engineering scaffolds. The pore sizes of the fabricated PCHPs were evaluated using ImageJ analysis, which showed a progressive reduction in pore size as the Gel-MA concentration increased from 2.5% to 4.5% (w/v) (Figure 2d).

Further, PCHPs porosity was quantified using the ethanol infiltration method. As shown in Figure 2e, the PCHPs exhibited a progressive reduction in porosity with increasing Gel-MA concentration from 2.5% to 4.5% (w/v). Both SEM and porosity results demonstrate high crosslinking efficiency and a highly interconnected pore network, which promotes the transport of minerals and other bioactive components to the injured microenvironment. Here, a porous structure will facilitate cell adhesion, proliferation, and nutrient flow between the skin and its surrounding environment, thereby supporting adhesion to the skin (Mukasheva et al., 2024; Khan et al., 2024).

### 3.4 Swelling and degradation behavior

We analyzed the swelling ratio of PCHPs in DMEM cell culture media. All the PCHPs showed an increase in swelling ratio over time, reaching equilibrium after 4–5 h. The swelling ratio decreased with increasing Gel-MA concentration, as the water uptake was observed to be highest with PCHP-2.5K/2.5G and lowest with PCHP-2.5K/4.5G due to the decreased pore size (Figure 2f). These results are consistent with SEM studies, which support higher swelling behavior with PCHP-2.5K/2.5G due to the presence of comparatively large pores. The obtained swelling supports the hydrogel’s capacity to absorb exudates (fluids) from the wound area, thereby reducing frequent microbial invasion and facilitating faster healing of chronic wounds (Lan et al., 2021).

PCHPs were also tested for degradation in DMEM at 37 °C to quantify polymer degradation and assess biodegradation. We measured the PCHP’s weight loss at different time points from day 1 to day 15. The obtained graph of weight loss vs. time showed a slow degradation of 15%–30% of PCHP films within 15 days (Figure 2g). The remaining undegraded PCHP films showed higher stability in DMEM. These results indicate that the visible-light photocrosslinking strategy is efficient and suitable for fabricating stable constructs for tissue engineering, thereby enhancing cell-scaffold interactions, migration, proliferation, and nutrient transport into the tissue environment through its swelling and degradation profiles (Mndlovu et al., 2024).

### 3.5 Viscoelastic behavior of the PCHPs

**FIGURE 3:**
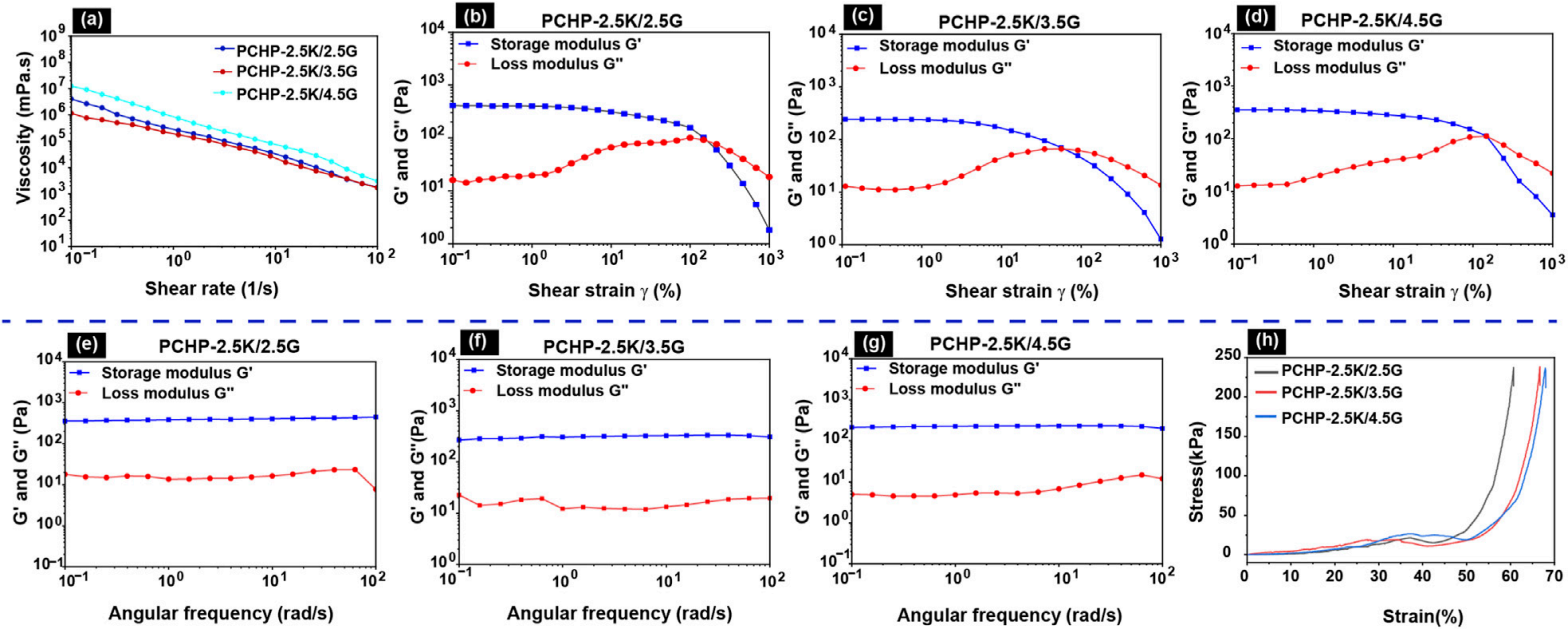
Rheological and mechanical characterization of PCHPs. **(a)** Viscosity as a function of shear rate, illustrating the shear-thinning behavior of the hydrogels. Amplitude sweep measurements presenting the storage modulus (G′) and loss modulus (G″) for **(b)** PCHP-2.5K/2.5G, **(c)** PCHP-2.5K/3.5G, and **(d)** PCHP-2.5K/4.5G. Frequency sweep measurements performed within the linear viscoelastic region (LVR) for **(e)** PCHP-2.5K/2.5G, **(f)** PCHP-2.5K/3.5G, and **(g)** PCHP-2.5K/4.5G. **(h)** Compressive stress-strain curves and corresponding mechanical properties of different PCHPs.

We analyzed the rheological properties to understand the behavior and viscoelastic performance of PCHPs. Viscosity properties at different shear rates from 10^−2^ to 10^2^ at room temperature were evaluated to know the shear-thinning behavior. The viscosity measurement of the PCHPs at various shear rates revealed increased shear-thinning properties with increasing shear rate (Figure 3a). The shear-thinning behavior of the PCHPs enables them for multiple biomedical applications, particularly for 3D printing. Additionally, we investigated the rheological properties of the PCHPs through amplitude sweep measurement. Plot of PCHPs depicted the gel-to-sol transition region, occurring around a strain of 120%, marked by the crossover of G' (storage modulus) and G'' (loss modulus), which demonstrates a shift in the viscoelastic behavior of PCHP (Figures 3b–d). Furthermore, the frequency sweep measurement of PCHPs was conducted within the linear viscoelastic region (0.1–100 rad/s) at a constant strain of 0.5% to evaluate its viscoelastic stability, revealing a higher storage modulus (G′) than the loss modulus (G″), indicating a dominant elastic behavior (Figures 3e–g). These viscoelastic characteristics support the application of PCHPs in biomedical applications, particularly for biological tissue mimicking and cytocompatibility (Chaudhuri, 2017; Sawadkar et al., 2025).

### 3.6 Mechanical performance of the PCHPs

To use a particular hydrogel or matrix for any tissue engineering application, mechanical properties are a crucial criterion to be analysed, as different tissues exhibit varying strengths. Based on the tissue of interest, scaffolds need to be fabricated because they influence cell-cell and cell-scaffold interactions and should mimic the ECM, thereby supporting cell migration and the exchange of micro- and macro-particles to repair the tissue’s defective environment. The load-bearing capacity of the PCHPs was analysed using a compression test with a universal testing machine. The stress–strain curve indicated that as the strain increased, the stress also increased, with the yield point occurring between 220 and 250 kPa (Figure 3h). All the PCHPs exhibited a J-shaped stress-strain plateau, which is characteristic of many biological tissues. Nonlinear mechanical properties are a distinctive feature of soft tissues and are highly desirable for scaffold-based wound dressings. J-shaped stress-strain behavior provides high stretchability, prevents tissues from experiencing excessive strain, and good extendability, collectively providing mechanical resistance to deformation. Importantly, wounds in movable parts, such as the elbow, knee, and wrist, often delay healing due to frequent movement, so scaffolds used for wound healing should be stretchable, adhesive, and function under dynamic conditions (Todros et al., 2023). The stress-strain curves of the PCHPs showed increased strain with increasing stress. The compressive moduli were 238 kPa for PCHP-2.5K/2.5G, 239 kPa for PCHP-2.5K/3.5G, and 234 kPa for PCHP-2.5K/4.5G. These values are similar to those of other hydrogel-based wound dressings and fall within the lower range of mechanical properties for native skin tissues, especially the dermal layer (approximately 88–300 kPa) (Levental et al., 2007; Li et al., 2012; Goswami et al., 2026). Such mechanical characteristics are advantageous for wound-dressing applications, as they provide adequate structural integrity while preserving the flexibility necessary to conform to irregular wound surfaces. These findings indicate that the developed hydrogels possess sufficient mechanical robustness to endure handling and application, while maintaining the flexibility required for effective wound coverage.

### 3.7 Antimicrobial studies

**FIGURE 4:**
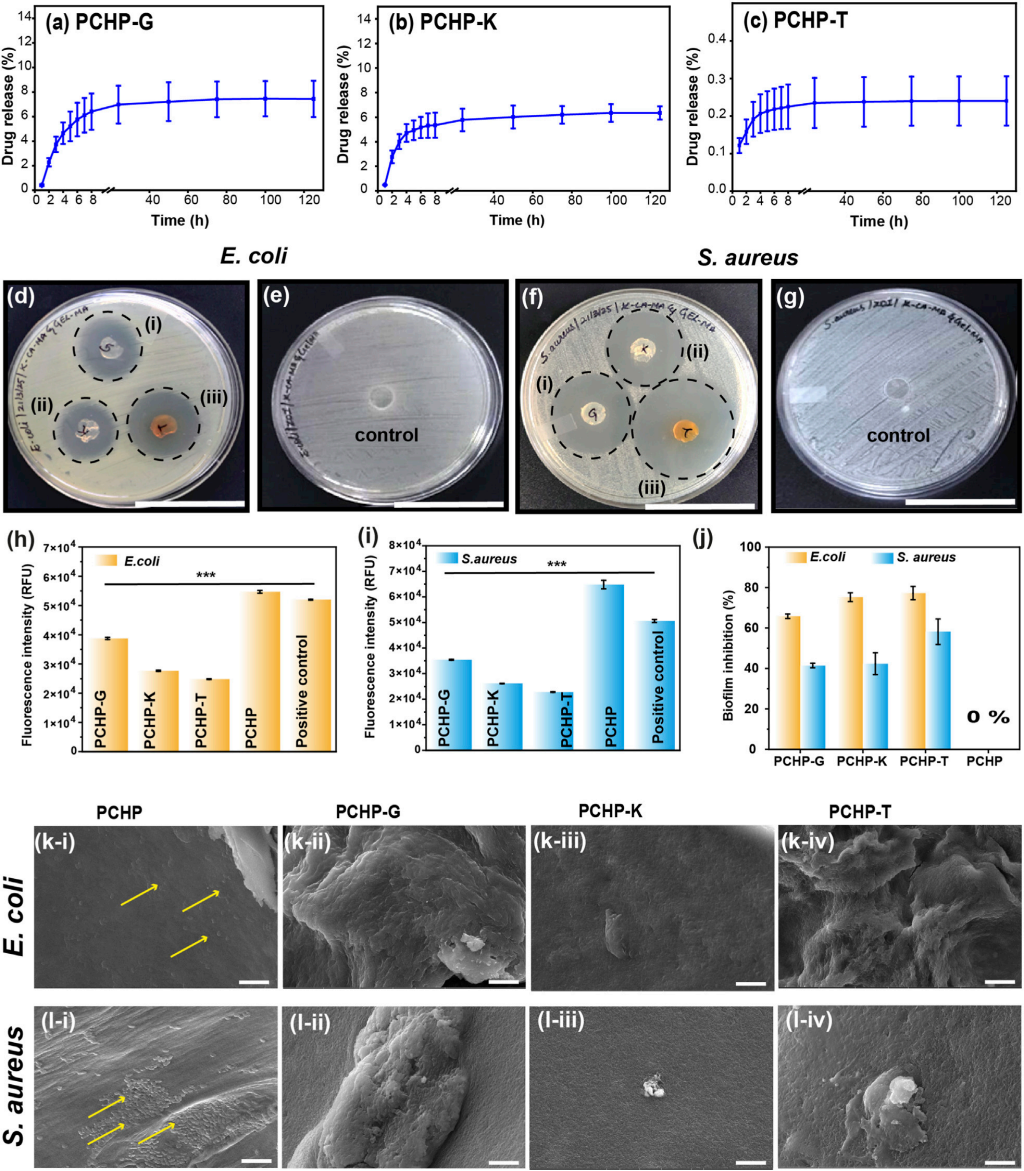
Time-dependent release profile of **(a)** gentamycin, **(b)** kanamycin, and **(c)** tetracycline from PCHP-2.5K/2.5G in PBS (10 mM, pH 7.5) at 37 °C. Digital images of agar plates showing the zone of inhibition for the samples of PCHP, PCHP-G, PCHP-K, and PCHP-T, and PCHP as a control against **(d,e)** *E.coli* and **(f,g)** *Staphylococcus aureus*. Scale bar for agar plates: 45 mm. Alamar assay showing the quantification of **(h)** *Escherichia coli*, and **(i)** *Staphylococcus aureus* cell attachment on different PCHP samples by measuring the fluorescence intensity (excitation at 560 nm, emission at 590 nm). **(j)** Antibiofilm activity was measured using the crystal violet biofilm assay against *Escherichia coli* and *Staphylococcus aureus* cells on the samples. Data are presented as mean ± SD (n = 3). Statistical significance was defined as ***p < 0.001. SEM images showing the bacterial cell attachment of **(k)** *Escherichia coli* and **(l)** *Staphylococcus aureus* cells on control samples of (k-i and l-i) PCHP, while no detectable bacterial attachment was observed on the antibiotic-loaded samples, namely, (k-ii and l-ii) PCHP-G, (k-iii and l-iii) PCHP-K, and (k-iv and l-iv) PCHP-T. Scale bar: 5 µm.

One significant research challenge in treating chronic wounds is that they often become infected with bacteria, which hinders wound healing. *E. coli* (*Escherichia coli*) and *S. aureus* (*Staphylococcus aureus)* are common bacteria that frequently infect wounds. To impart antibacterial properties, the PCHP-2.5K/2.5G formulation was selected for antibiotic loading with gentamicin (G), kanamycin (K), and tetracycline (T) via a physical entrapment method. The cumulative *in vitro* drug release of gentamycin, kanamycin and tetracycline from the hydrogels were quantified using UV-Visible spectrophotometry. Release profiles of the encapsulated antibiotics from all the PCHPs (PCHP-G, PCHP-K, and PCHP-T) are shown in Figures 4a–c. Over 125 h, 7.4% of gentamicin, 6.3% of kanamycin, and 0.25% of tetracycline were released in PBS at 37 °C. These data demonstrate an initial rapid release of the antibiotics, followed by a slower, sustained release phase. This release pattern results from the entrapment of antibiotics within the hydrogel matrix, thereby facilitating controlled, prolonged drug delivery. The initial burst release is essential for addressing bacterial invasion in the wound microenvironment, while the subsequent sustained release may prevent further infection. These findings indicate that this patch system is a promising controlled, localized drug-delivery platform for chronic wound treatment.

The antibacterial efficacy of the hydrogels was evaluated against *E. coli* and *S. aureus*, with PCHPs lacking antibiotics serving as the control. All antibiotic-loaded PCHPs (PCHP-G, PCHP-K, and PCHP-T) demonstrated significant zones of inhibition (ZOI) against both *E. coli* (Figure 4d) and *S. aureus* (Figure 4f), indicating superior bacterial inhibition compared to the control (Figures 4e,g). These findings suggest their suitability for use in wound environments to prevent bacterial infections that impede wound healing. Antibacterial activity was further quantified by measuring the colony-forming units (CFUs) of antibiotic-loaded PCHPs against *E. coli* and *S. aureus*. CFU counts on agar plates confirmed the antibacterial activity of PCHP-G, PCHP-K, and PCHP-T, highlighting their potential for sustained, localized antibiotic release at the wound site. The CFU values for E*. coli* with PCHP-G, PCHP-K, and PCHP-T were 7.9 × 10^4^, 7.8 × 10^4^, and 7.1 × 10^4^ CFU/mL, respectively. For *S. aureus*, the CFU count for tetracycline was 1.26 × 10^5^, whereas no colonies were observed for the other drugs (Gou et al., 2024).

Bacterial cell viability was further assessed using Alamar Blue, a cell-permeable reagent that measures viability by reduction of the non-fluorescent blue dye to a fluorescent pink via metabolic activity. As shown in Figures 4h,i, incubation of *E. coli* and *S. aureus* cultures with the antibiotic-loaded hydrogels PCHP-G, PCHP-K, and PCHP-T resulted in a significant reduction in metabolic activity compared to both the control and antibiotic-free PCHP. The observed decrease in fluorescence intensity for PCHP-G, PCHP-K, and PCHP-T indicates reduced bacterial viability attributable to the antibacterial effect of the incorporated antibiotics. Among these, PCHP-T exhibited the highest antibacterial activity and the lowest fluorescence intensity.

Another major issue in wound healing is biofilm formation, which refers to the aggregation of bacteria on the surface of the infected wound and their encasement within a self-produced extracellular polymeric matrix. Further biofilm formation delays various cellular mechanisms and increases the mortality and morbidity. In this study, antibiotics were incorporated into PCHPs to inhibit bacterial attachment and biofilm growth, which remains a major challenge in the biomedical field. We assessed the antibiofilm activity of PCHP-G, PCHP-K, and PCHP-T using a crystal violet biofilm assay against Gram-negative (*E. coli*) and Gram-positive (*S. aureus*) bacteria: As shown in Figure 4j, PCHP without an antibiotic showed no detectable biofilm inhibition (0%), indicating that the patch alone lacks inherent antibiofilm activity. The PCHP-G, PCHP-K, and PCHP-T showed superior antibiofilm activity, with PCHP-T achieving the highest biofilm-inhibition rates (77.28% and 58.2%) against *E. coli* and *S. aureus,* respectively. As tetracycline has broad-spectrum activity, it shows superior antibiofilm activity and strong bacteriostatic effects, destabilizing bacteria and preventing attachment and biofilm formation. Further, PCHP-K exhibited substantial antibiofilm activity, with 75.3% and 42.32% inhibition of *E. coli* and *S. aureus*, respectively, whereas PCHP-G demonstrated 65.83% and 41.48% inhibition of *E. coli* and *S. aureus*, respectively. The result confirms that the release of loaded antibiotics is sustainable and inhibits bacterial biofilm formation through their permeability. As *S. aureus* has a thicker, denser peptidoglycan layer, it shows reduced biofilm and reduced drug diffusion into the biofilm.

Further SEM was used to investigate bacterial cell attachment to the PCHPs following antibiotic encapsulation. As shown in Figures 4k–i, and l-i, antibiotics-free PCHPs showed bacterial cell attachment, dense colonization, and displayed intact cell morphology of *E. coli* and *S. aureus*, indicating normal bacterial growth. In contrast, Figures 4k,l, antibiotic-loaded hydrogels, namely, PCHP-G (k-ii, l-ii), PCHP-K (k-iii, l-iii), and PCHP-T (k-iv, l-iv), showed no bacterial cell attachment, likely due to sustained antibiotic release into the surrounding medium. Overall, the results confirm that incorporating antibiotics into PCHPs enhances its antibiofilm efficacy and can be used as an advanced wound dressing material to prevent polymicrobial-associated infections (Min et al., 2020; Anjum et al., 2018).

### 3.8 *In vitro* antioxidant and cytocompatibility studies

**FIGURE 5:**
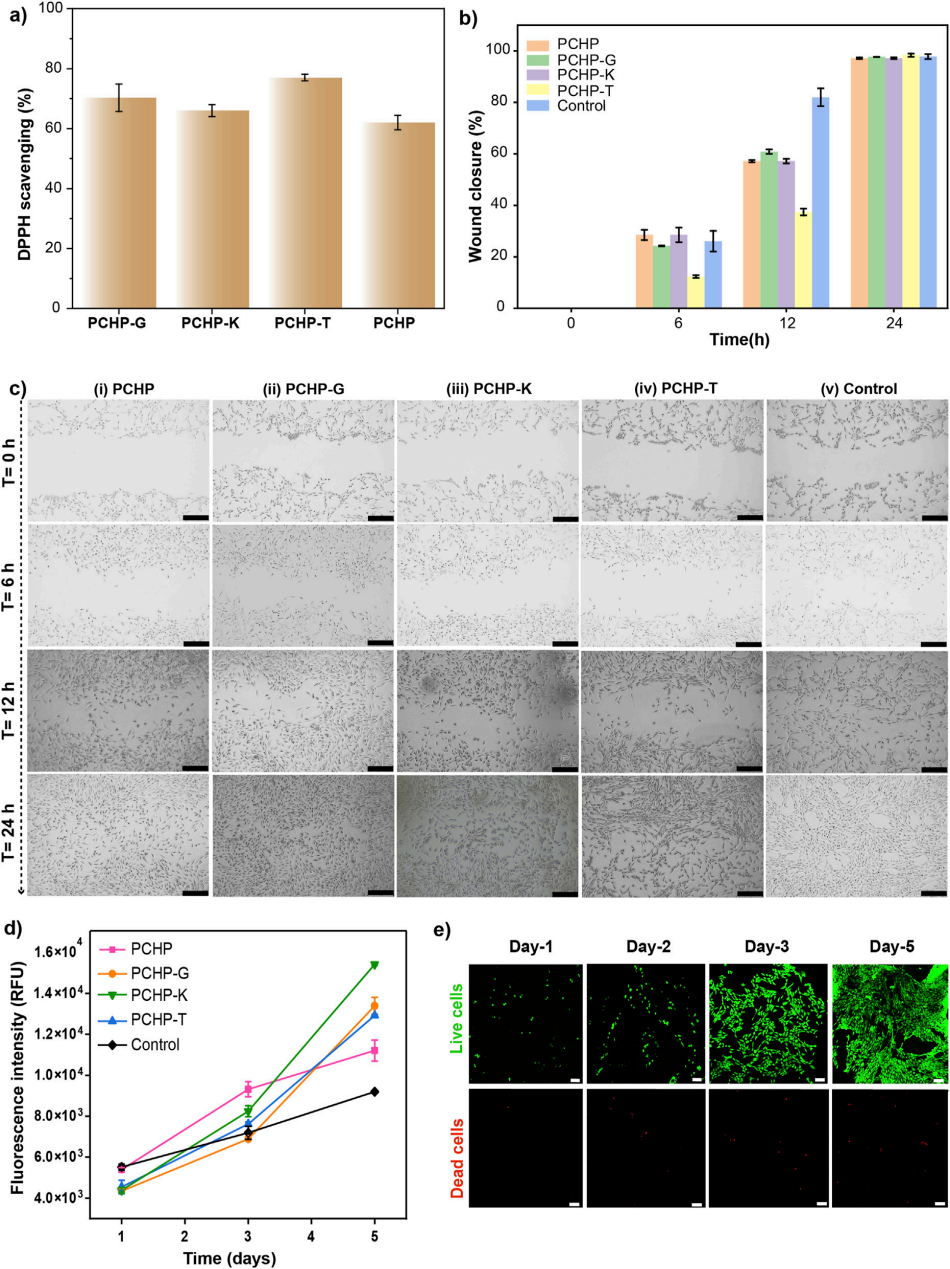
**(a)** DPPH radical scavenging activity of drug-encapsulated PCHP. **(b)** Quantitative analysis of scratch closure (%) of NIH3T3 fibroblast cells cultured in media extracts of the antibiotic-loaded PCHP. Data are presented as mean ± SD (n = 3). **(c)** Representative bright-field microscopic images of NIH3T3 cells cultured on a 35 mm cell culture plate containing media extracts from (i) PCHP, (ii) PCHP-G, (iii) PCHP-K, (iv) PCHP-T, and (v) control at different incubation time points. Scale bars: 100 µm. **(d)** Alamar blue assay data showing the proliferation of NIH3T3 cultured on the PCHP with and without antibiotics. At each daily time point (days 1, 3, and 5), the media were removed, and the cells were incubated with 10% (v/v) Alamar Blue reagent prepared in cell culture medium for 3 h. Fluorescence was measured to quantify the metabolic conversion of resazurin (blue) to resorufin (pink) at excitation/emission wavelengths of 560 nm and 590 nm, respectively. **(e)** Confocal laser fluorescence microscopy images of NIH3T3 cells cultured on PCHP at days 1, 2, 3, and 5, stained with calcein (live, green) and ethidium homodimer (dead, red). Scale bars: 100 µm. Data are presented as mean ± SD (n = 3).

Various medical conditions, such as chronic wounds, osteoarthritis, and other diseases, are associated with elevated oxidative stress due to excessive free radicals. Free radicals are highly reactive molecules with unpaired electrons that react with nearby biomolecules to stabilize themselves. However, excess production induces oxidative stress in the cellular components, leading to severe pathological conditions. Therefore, scavenging excessive free radicals is crucial for efficient wound healing. We evaluated the antioxidant activity of drug-encapsulated PCHPs using the DPPH assay, and the results demonstrated significant antioxidant activity across all formulated scaffolds (Figure 5a). The tetracycline-loaded patch showed the highest antioxidant activity (77%), followed by gentamicin (70.3%), kanamycin (66%), and the drug-free hydrogel (62%). Which still exhibited substantial scavenging activity (62%). The results demonstrate that the formulated patch exhibits strong antioxidant activity, which may facilitate faster wound healing (Tran et al., 2020).

Further, a basic scratch assay was conducted *in vitro* to simulate the wound environment and evaluate cell migration and healing capacity. Optical microscopy demonstrated a reduction in the scratch area and increased cell migration over time. Quantitative data in Figure 5b and microscopy images in Figure 5c confirm that extracts from antibiotic-loaded PCHPs promote cell migration and proliferation, effectively facilitating *in vitro* wound closure by replicating physiological cell-cell interactions. The progressive closure of the scratch area supports the potential application of PCHPs in wound healing.

*In vitro* cytocompatibility studies were performed on NIH3T3 fibroblasts, a well-established cell line for skin substitute testing, to understand cell-scaffold interactions for wound-healing applications. An alamar blue assay was conducted to determine the cytotoxicity of PCHP-2.5K/2.5G loaded with antibiotics against NIH3T3 fibroblasts. Alamar blue contains a non-fluorescent blue dye, resazurin, which viable cells convert into a pink, highly fluorescent resorufin dye. We measured fluorescence intensity to correlate cell viability and proliferation in PCHP-2.5K/2.5G loaded with or without antibiotics from day 1 to day 5. PCHPs loaded with antibiotics clearly showed increased fluorescence intensity over time, indicating good cell viability and proliferation of NIH3T3 cells from day 1–5, compared to the control 2D cell culture plate (Figure 5d). The obtained data are consistent with previously reported literature, which indicates that scaffolds loaded with antibiotics enhance tissue regeneration at the wound site by facilitating ECM remodeling for effective wound healing. Over the same time intervals, cell viability was analyzed using a live-dead assay performed on PCHP-2.5K/2.5G. On each day of analysis, PCHPs encapsulated with cells were stained with calcein AM and ethidium homodimer to distinguish viable from dead cell populations (Figure 5e). Ethidium homodimer is a membrane-impermeable dye that stains dead cells by binding to their nucleic acids and emitting a red fluorescent color. In contrast, calcein AM is a permeable dye that enters live cells and is converted to free fluorescent calcein by intercellular esterases. Fluorescence images taken at different times clearly show a higher number of viable cells (green fluorescence) than dead cells (red fluorescence) from day 1 to day 5, demonstrating the cytocompatibility of PCHP-2.5K/2.5G for cell attachment and proliferation.

### 3.9 DLP printing

**FIGURE 6:**
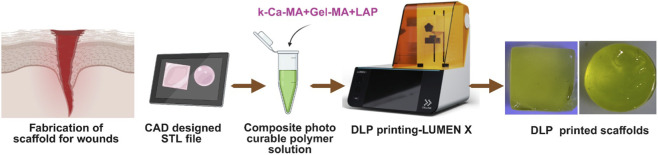
Schematic illustration of the DLP printing process used for fabricating 3D scaffolds employing photocurable κ-Ca-MA and Gel-MA biopolymers.

In this study, we used a DLP printer to fabricate a patch composed of PCHP-2.5K/2.5 G. The DLP printing technique was developed to fabricate photocurable materials that closely mimic the complex structures of tissues and organs. The DLP printer is an efficient, time-saving, high-resolution printing technique for 3D printing organs and tissues. Using DLP printing, various in-built structures were printed with 0.4% LAP (photo initiator) and 0.3 mM of (photo blocker). Here, scaffolds were printed with high fidelity in two dimensions (8 × 8 × 1.8 mm and 8 × 8 × 3 mm) as squares and circles, with 10 and 20 layers, respectively (Figure 6). Each layer was photocured for 20 s using UV-C irradiation (402 nm). 3D-printed scaffolds made from the new photocurable composite polymer will be promising tissue models for wound-healing applications.

## 4 Discussion

Nowadays, carbohydrate-based biopolymers are widely adopted in soft tissue engineering research owing to their biocompatibility and non-cytotoxicity properties, as well as their ability to replicate the native extracellular matrix (ECM) (Tchobanian et al., 2019). In the current study, the selected carbohydrate-based biopolymer, κ-Ca, is derived from marine red seaweed and has been applied across multiple biomedical, food, and pharmaceutical industries. κ-Ca exhibits good gelling properties, demonstrates biocompatibility, tunable viscosity, biodegradability, non-cytotoxicity, and resembles natural glycosaminoglycans (GAGs), which bio-mimic the ECM (Neamtu et al., 2022). Another protein-based biopolymer used in the current research is gelatin, which is widely used in the pharmaceutical, regenerative medicine, biomedical, and food industries. The gelatin exhibits good bioavailability and compatibility, and the presence of the RGD (arginine-glycine-aspartic acid) group supports cellular activity, which, in turn, helps regenerate the affected tissue environment more quickly than other polymers (Alipal et al., 2021).

The selected biopolymers were chemically functionalized with a photocurable functional group, methacrylic anhydride, to enable photo-crosslinking under visible light (420 nm). These functionalized biopolymers were then used to fabricate hydrogel patch films. As shown in the results, photocrosslinking of κ-Ca-MA and Gel-MA promotes strong intra- and intermolecular crosslinking, improving structural integrity while maintaining biocompatibility, making them suitable for applications in regenerative medicine. The porosity of these materials can be tuned by varying the polymer amounts, depending on the specific application. This tunable porosity, slow degradation, and ECM-mimicking properties will facilitate faster healing of chronic wounds by providing a desired microenvironment for cell attachment and promoting vascularization over time. To minimize the risk of infection, we incorporated antibiotics into PCHPs to provide sustained, localized release at the wound site, thereby creating an optimal environment for cell proliferation and tissue remodeling, which helps overcome impairments in the healing process. The outcomes of the current research work, including shear-thinning gel behavior, sustained swelling, slow degradation, non-cytotoxicity, high cell viability of encapsulated cells, and printability, demonstrate the potential of PCHP as a viable alternative to skin grafts.

## 5 Conclusion

In summary, we fabricated a hydrogel patch using photocurable polymers κ-Ca-MA and Gel-MA, and a photocrosslinking approach to develop PCHP for wound healing and recovery. The synthesized hydrogel patch was analyzed for its swelling and degradation characteristics. The results demonstrate good swelling properties and slow degradation, which will facilitate the removal of wound exudates and sustain drug delivery at the wound area upon application. The rheological and mechanical analyses revealed shear-thinning behavior, a high storage modulus, viscoelasticity, and mechanical stability. Antibiotics encapsulated in PCHP-2.5K/2.5G exhibited slow, sustained release and promising antimicrobial activity against *E. coli* and *S. aureus*, thereby enhancing wound-healing capability by combining physical wound protection with antimicrobial action. Antioxidant and scratch assays yielded promising results, supporting the potential for wound-healing applications. The fibroblast cells seeded onto PCHP-2.5K/2.5G exhibited higher cell attachment, viability, non-cytotoxicity, and proliferation over 5 days than on the control scaffold. Additionally, using light-based DLP printing, 3D-printed PCHP-2.5K/2.5G were fabricated to ensure usability for patient-specific applications. Ultimately, by consolidating all the features demonstrated throughout this work, the fabricated PCHP can be assessed for its efficiency in soft tissue engineering applications, primarily for chronic wound healing.

## Data Availability

The original contributions presented in the study are included in the article/supplementary material, further inquiries can be directed to the corresponding author.

## References

[B1] Alipal J. Pu'ad Nas M. Lee T. C. Nayan N. H. M. Sahari N. Basri H. (2021). A review of gelatin: properties, sources, process, applications, and commercialisation. Mater. Today Proc. 42, 240–250. 10.1016/j.matpr.2020.12.922

[B2] Anjum A. Sim C. H. Ng S. F. (2018). Hydrogels containing antibiofilm and antimicrobial agents beneficial for biofilm-associated wound infection: formulation characterizations and *in vitro* study. AAPS PharmSciTech 19 (3), 1219–1230. 10.1208/s12249-017-0937-4 29280044

[B3] Chakraborty A. Alexander S. Luo W. Al-Salam N. Van Oirschot M. Ranganath S. H. (2023). Engineering multifunctional adhesive hydrogel patches for biomedical applications. Interdiscip. Med. 1 (4), e20230008. 10.1002/inmd.20230008

[B4] Chaudhuri O. (2017). Viscoelastic hydrogels for 3D cell culture. Biomaterials Sci. 5 (8), 1480–1490. 10.1039/c7bm00261k 28584885

[B5] Chen S. Wang Y. Lai J. Tan S. Wang M. (2023). Structure and properties of gelatin methacryloyl (GelMA) synthesized in different reaction systems. Biomacromolecules 24 (6), 2928–2941. 10.1021/acs.biomac.3c00302 37212876

[B6] Choi J. R. Yong K. W. Choi J. Y. Cowie A. C. (2019). Recent advances in photo-crosslinkable hydrogels for biomedical applications. Biotechniques 66 (1), 40–53. 10.2144/btn-2018-0083 30730212

[B7] Deng X. Gould M. Ali M. A. (2022). A review of current advancements for wound healing: biomaterial applications and medical devices. J. Biomed. Mater Res. B Appl. Biomater. 110 (11), 2542–2573. 10.1002/jbm.b.35086 35579269 PMC9544096

[B8] Echave M. C. Saenz del Burgo L. Pedraz J. L. Orive G. (2017). Gelatin as biomaterial for tissue engineering. Curr. Pharm. Des. 23 (24), 3567–3584. 10.2174/0929867324666170511123101 28494717

[B9] Elango J. Zamora-Ledezma C. Maté-Sánchez de Val J. E. (2023). Natural vs synthetic polymers: how do they communicate with cells for skin regeneration—A review. J. Compos. Sci. 7 (9), 385. 10.3390/jcs7090385

[B10] Fasiku V. O. Omolo C. A. Devnarain N. Ibrahim U. H. Rambharose S. Faya M. (2021). Chitosan-based hydrogel for the dual delivery of antimicrobial agents against bacterial methicillin-resistant *Staphylococcus aureus* biofilm-infected wounds. ACS Omega 6 (34), 21994–22010. 10.1021/acsomega.1c02547 34497894 PMC8412894

[B11] Freedman B. R. Hwang C. Talbot S. Hibler B. Matoori S. Mooney D. J. (2023). Breakthrough treatments for accelerated wound healing. Sci. Adv. 9 (20), eade7007. 10.1126/sciadv.ade7007 37196080 PMC10191440

[B12] Gobi R. Ravichandiran P. Babu R. S. Yoo D. J. (2021). Biopolymer and synthetic polymer-based nanocomposites in wound dressing applications: a review. Polym. (Basel) 13 (12), 1962. 10.3390/polym13121962 34199209 PMC8232021

[B13] Golshirazi A. Labbaf S. Varshosaz J. (2025). Optimised GelMA/Tragacanth gum hydrogel loaded with vanillic acid for biomedical applications. Int. J. Biol. Macromol. 311, 143535. 10.1016/j.ijbiomac.2025.143535 40294682

[B14] Goswami A. K. Giduturi V. K. Yerramilli S. N. Chauhan V. S. Yadav N. (2026). Tissue engineering: hydrogel scaffolds and mechanical properties as key design parameters. Adv. Colloid Interface Sci. 347, 103691. 10.1016/j.cis.2025.103691 41125016

[B15] Gou Y. Hu L. Liao X. He J. Liu F. (2024). Advances of antimicrobial dressings loaded with antimicrobial agents in infected wounds. Front. Bioeng. Biotechnol. 12, 1431949. 10.3389/fbioe.2024.1431949 39157443 PMC11327147

[B16] Gounden V. Singh M. (2024). Hydrogels and wound healing: current and future prospects. Gels 10 (1), 43. 10.3390/gels10010043 38247766 PMC10815795

[B17] Gruppuso M. Turco G. Marsich E. Porrelli D. (2021). Polymeric wound dressings, an insight into polysaccharide-based electrospun membranes. Appl. Mater. Today 24, 101148. 10.1016/j.apmt.2021.101148

[B18] Hachim D. Whittaker T. E. Kim H. Stevens M. M. (2019). Glycosaminoglycan-based biomaterials for growth factor and cytokine delivery: making the right choices. J. Control. Release 313, 131–147. 10.1016/j.jconrel.2019.10.018 31629041 PMC6900262

[B19] Hudzicki J. (2009). Kirby-bauer disk diffusion susceptibility test protocol. Am. Society Microbiology 15 (1), 1–23.

[B20] Jabeen F. Zil e A. Ahmad R. Mir S. Awwad N. S. Ibrahium H. A. (2025). Carrageenan: structure, properties and applications with special emphasis on food science. RSC Adv. 15 (27), 22035–22062. 10.1039/d5ra03296b 40584756 PMC12203331

[B21] Jaiswal R. Sherje A. P. (2024). Recent advances in biopolymer-based smart hydrogel for wound healing. J. Drug Deliv. Sci. Technol. 99, 105990. 10.1016/j.jddst.2024.105990

[B22] Khan R. Aslam Khan M. U. Stojanović G. M. Javed A. Haider S. Abd Razak S. I. (2024). Fabrication of bilayer nanofibrous-hydrogel scaffold from bacterial cellulose, PVA, and gelatin as advanced dressing for wound healing and soft tissue engineering. ACS Omega 9 (6), 6527–6536. 10.1021/acsomega.3c06613 38371763 PMC10870282

[B23] Kowsari K. Lee W. Yoo S.-S. Fang N. X. (2021). Scalable visible light 3D printing and bioprinting using an organic light-emitting diode microdisplay. iScience 24 (11), 103372. 10.1016/j.isci.2021.103372 34825139 PMC8605192

[B24] Kumari S. Mondal P. Chatterjee K. (2022). Digital light processing-based 3D bioprinting of κ-carrageenan hydrogels for engineering cell-loaded tissue scaffolds. Carbohydr. Polym. 290, 119508. 10.1016/j.carbpol.2022.119508 35550782

[B25] Kumari S. Mondal P. Tyeb S. Chatterjee K. (2024). Visible light-based 3D bioprinted composite scaffolds of κ-carrageenan for bone tissue engineering applications. J. Mater. Chem. B 12 (7), 1926–1936. 10.1039/d3tb02179c 38314524

[B26] Lan G. Zhu S. Chen D. Zhang H. Zou L. Zeng Y. (2021). Highly adhesive antibacterial bioactive composite hydrogels with controllable flexibility and swelling as wound dressing for full-thickness skin healing. Front. Bioeng. Biotechnol. 9, 785302. 10.3389/fbioe.2021.785302 35004645 PMC8735859

[B27] Levental I. Georges P. C. Janmey P. A. (2007). Soft biological materials and their impact on cell function. Soft Matter 3 (3), 299–306. 10.1039/b610522j 32900146

[B28] Li C. Guan G. Reif R. Huang Z. Wang R. K. (2012). Determining elastic properties of skin by measuring surface waves from an impulse mechanical stimulus using phase-sensitive optical coherence tomography. J. R. Soc. Interface 9 (70), 831–841. 10.1098/rsif.2011.0583 22048946 PMC3306653

[B29] Li S. Wang X. Chen J. Guo J. Yuan M. Wan G. (2022). Calcium ion cross-linked sodium alginate hydrogels containing deferoxamine and copper nanoparticles for diabetic wound healing. Int. J. Biol. Macromol. 202, 657–670. 10.1016/j.ijbiomac.2022.01.080 35066024

[B30] Li H. Dai J. Wang Z. Zheng H. Li W. Wang M. (2023). Digital light processing (DLP)-Based (bio)printing strategies for tissue modeling and regeneration. Aggregate 4 (2), e270. 10.1002/agt2.270

[B31] Liang C.-C. Park A. Y. Guan J.-L. (2007). *In vitro* scratch assay: a convenient and inexpensive method for analysis of cell migration *in vitro* . Nat. Protoc. 2 (2), 329–333. 10.1038/nprot.2007.30 17406593

[B32] Lin Z. Chen L. Qi C. Wang G. (2025). Skin microecology and skin barrier repair, wound healing and appendage regeneration. Regenes. Repair Rehabil. 1 (1), 1–5. 10.1016/j.rerere.2024.07.001

[B33] Liu L. Zhong X. Wang A. Gu Q. Sun C. Wang F. (2025). Biodegradable natural hydrogels: design, crosslinking, and medical applications. Mater. Today Commun. 46, 112929. 10.1016/j.mtcomm.2025.112929

[B34] Menon A. Elkhoury K. Zahraa A. Sapudom J. Rahic Z. Gunsalus K. C. (2024). Digital light processing 3D printing of dual crosslinked meniscal scaffolds with enhanced physical and biological properties. Adv. Compos. Hybrid Mater. 8 (1), 92. 10.1007/s42114-024-01196-8

[B35] Mihaila S. M. Gaharwar A. K. Reis R. L. Marques A. P. Gomes M. E. Khademhosseini A. (2013). Photocrosslinkable kappa-carrageenan hydrogels for tissue engineering applications. Adv. Healthc. Mater 2 (6), 895–907. 10.1002/adhm.201200317 23281344

[B36] Min J. G. Sanchez Rangel U. J. Franklin A. Oda H. Wang Z. Chang J. (2020). Topical antibiotic elution in a collagen-rich hydrogel successfully inhibits bacterial growth and biofilm formation *in vitro* . Antimicrob. Agents Chemother. 64, e00136-20. 10.1128/AAC.00136-20 32690648 PMC7508589

[B37] Mndlovu H. Kumar P. du Toit L. C. Choonara Y. E. (2024). A review of biomaterial degradation assessment approaches employed in the biomedical field. Npj Mater. Degrad. 8 (1), 66. 10.1038/s41529-024-00487-1

[B38] Moon S. H. Hwang H. J. Jeon H. R. Park S. J. Bae I. S. Yang Y. J. (2023). Photocrosslinkable natural polymers in tissue engineering. Front. Bioeng. Biotechnol. 11, 11–2023. 10.3389/fbioe.2023.1127757 36970625 PMC10037533

[B39] Mukasheva F. Adilova L. Dyussenbinov A. Yernaimanova B. Abilev M. Akilbekova D. (2024). Optimizing scaffold pore size for tissue engineering: insights across various tissue types. Front. Bioeng. Biotechnol. 12, 1444986. 10.3389/fbioe.2024.1444986 39600888 PMC11588461

[B40] Neamtu B. Barbu A. Negrea M. O. Berghea-Neamțu C. Popescu D. Zăhan M. (2022). Carrageenan-based compounds as wound healing materials. Int. J. Mol. Sci. 23 (16), 9117. 10.3390/ijms23169117 36012381 PMC9409225

[B41] Pan H. Fan D. Cao W. Zhu C. Duan Z. Fu R. (2017). Preparation and characterization of breathable hemostatic hydrogel dressings and determination of their effects on full-thickness defects. Polymers 9 (12), 727. 10.3390/polym9120727 30966027 PMC6418977

[B42] Pourmadadi M. Poorkhalili P. Ajalli N. Rahdar A. Pandey S. (2025). Next-generation antibacterial agents and advanced fabrication techniques for wound dressings: a multifunctional approach to accelerate healing. J. Drug Deliv. Sci. Technol. 111, 107206. 10.1016/j.jddst.2025.107206

[B43] Sawadkar P. Lali F. Garcia-Gareta E. Garrido B. G. Chaudhry A. Matharu P. (2025). Innovative hydrogels in cutaneous wound healing: current status and future perspectives. Front. Bioeng. Biotechnol. 13, 1454903. 10.3389/fbioe.2025.1454903 40421113 PMC12104307

[B44] Sheokand B. Vats M. Kumar A. Srivastava C. M. Bahadur I. Pathak S. R. (2023). Natural polymers used in the dressing materials for wound healing: past, present and future. J. Polym. Sci. 61 (14), 1389–1414. 10.1002/pol.20220734

[B45] Sonwane S. Bonde S. Bonde C. Chandarana C. (2025). Advances in gelatin-based scaffolds for tissue engineering applications: a review. J. Drug Deliv. Sci. Technol. 107, 106789. 10.1016/j.jddst.2025.106789

[B46] Sun M. Sun X. Wang Z. Guo S. Yu G. Yang H. (2018). Synthesis and properties of gelatin methacryloyl (GelMA) hydrogels and their recent applications in load-bearing tissue. Polym. 10 (11), 1290. 10.3390/polym10111290 30961215 PMC6401825

[B47] Tavakoli S. Kharaziha M. Kermanpur A. Mokhtari H. (2019). Sprayable and injectable visible-light Kappa-carrageenan hydrogel for *in-situ* soft tissue engineering. Int. J. Biol. Macromol. 138, 590–601. 10.1016/j.ijbiomac.2019.07.126 31344417

[B48] Tavakoli M. Labbaf S. Mirhaj M. Salehi S. Seifalian A. M. Firuzeh M. (2023). Natural polymers in wound healing: from academic studies to commercial products. J. Appl. Polym. Sci. 140 (22), e53910. 10.1002/app.53910

[B49] Tchobanian A. Van Oosterwyck H. Fardim P. (2019). Polysaccharides for tissue engineering: current landscape and future prospects. Carbohydr. Polym. 205, 601–625. 10.1016/j.carbpol.2018.10.039 30446147

[B50] Thi P. L. Lee Y. Tran D. L. Thi T. T. H. Kang J. I. Park K. M. (2020). *In situ* forming and reactive oxygen species-scavenging gelatin hydrogels for enhancing wound healing efficacy. Acta Biomater. 103, 142–152. 10.1016/j.actbio.2019.12.009 31846801

[B51] Todros S. Castilho M. Espino D. M. Pavan P. G. (2023). Editorial: mechanical behavior of hydrogels for soft tissue replacement and regeneration. Front. Bioeng. Biotechnol., 11. 1254076. 10.3389/fbioe.2023.1254076 37560535 PMC10408127

[B52] Tran D. L. Le Thi P. Hoang Thi T. T. Park K. D. (2020). Novel enzymatically crosslinked chitosan hydrogels with free-radical-scavenging property and promoted cellular behaviors under hyperglycemia. Prog. Nat. Sci. Mater. Int. 30 (5), 661–668. 10.1016/j.pnsc.2020.08.004

[B53] Uberoi A. McCready-Vangi A. Grice E. A. (2024). The wound microbiota: microbial mechanisms of impaired wound healing and infection. Nat. Rev. Microbiol. 22 (8), 507–521. 10.1038/s41579-024-01035-z 38575708

[B54] Waqar M. A. Mubarak N. Khan A. M. Shaheen F. Mustafa M. A. Riaz T. (2025). Recent advances in polymers, preparation techniques, applications and future perspectives of hydrogels. Int. J. Polym. Mater. Polym. Biomaterials 74 (4), 265–284. 10.1080/00914037.2024.2335163

[B55] Yan Y. Cao Y. Cheng R. Shen Z. Zhao Y. Zhang Y. (2022). Preparation and *in vitro* characterization of gelatin methacrylate for corneal tissue engineering. Tissue Eng. Regen. Med. 19 (1), 59–72. 10.1007/s13770-021-00393-6 34665455 PMC8782967

[B56] Zhang H. Lin X. Cao X. Wang Y. Wang J. Zhao Y. (2024). Developing natural polymers for skin wound healing. Bioact. Mater. 33, 355–376. 10.1016/j.bioactmat.2023.11.012 38282639 PMC10818118

